# PLD1 participates in BDNF-induced signalling in cortical neurons

**DOI:** 10.1038/srep14778

**Published:** 2015-10-06

**Authors:** Mohamed Raafet Ammar, Tamou Thahouly, André Hanauer, David Stegner, Bernhard Nieswandt, Nicolas Vitale

**Affiliations:** 1 Institut des Neurosciences Cellulaires et Intégratives (INCI), UPR-3212 Centre National de la Recherche Scientifique & Université de Strasbourg, 5 rue Blaise Pascal, 67084 Strasbourg, France; 2 Institut de Génétique et de Biologie Moléculaire et Cellulaire (IGBMC), Centre National de la Recherche Scientifique, Institut National de la Santé et de la Recherche Médicale & Université de Strasbourg, BP 10142, 67404 Illkirch Cedex, France; 3 University of Würzburg, Department of Experimental Biomedicine, University Hospital and Rudolf Virchow Center; Josef-Schneider-Straße 2; D15, 97080 Würzburg, Germany

## Abstract

The brain-derived neurotrophic factor BDNF plays a critical role in neuronal development and the induction of L-LTP at glutamatergic synapses in several brain regions. However, the cellular and molecular mechanisms underlying these BDNF effects have not been firmly established. Using *in vitro* cultures of cortical neurons from knockout mice for *Pld1* and *Rsk2,* BDNF was observed to induce a rapid RSK2-dependent activation of PLD and to stimulate BDNF ERK1/2-CREB and mTor-S6K signalling pathways, but these effects were greatly reduced in *Pld1*^−/−^ neurons. Furthermore, phospho-CREB did not accumulate in the nucleus, whereas overexpression of PLD1 amplified the BDNF-dependent nuclear recruitment of phospho-ERK1/2 and phospho-CREB. This BDNF retrograde signalling was prevented in cells silenced for the scaffolding protein PEA15, a protein which complexes with PLD1, ERK1/2, and RSK2 after BDNF treatment. Finally PLD1, ERK1/2, and RSK2 partially colocalized on endosomal structures, suggesting that these proteins are part of the molecular module responsible for BDNF signalling in cortical neurons.

Brain-derived neurotrophic factor (BDNF) is the most widely expressed and well-characterized member of the neurotrophin family in the mammalian brain. It is translated as a precursor protein (proBDNF), consisting of an N-terminal prodomain and a C-terminal mature domain. Mature BDNF is composed of dimers, and its effects are tightly regulated. BDNF can exert its functions in a highly localized manner, and also at a distance by anterograde or retrograde transport. Modest changes in BDNF levels affect the development and regulation of neural circuits and brain function. BDNF has been shown in cell culture to play an important role in neuronal survival and in the maintenance of most neuronal networks and is important for functional and structural synaptic plasticity^1^,^2^,^3^. The current notion is that during development BDNF is involved in regulating the fine-tuning of the cortical network by selectively enhancing dendritic growth in an activity-dependent manner^4^,^5^. Furthermore, dendritic spine density and morphology of mature primary hippocampal neurons are significantly influenced by BDNF^6^,^7^.

On the other hand, the *in vivo* role of BDNF has been very difficult to evaluate in the post-natal brain as *Bdnf*^*−/−*^ mice die shortly after birth^8^. A number of studies using different conditional gene targeted mouse lines and Cre-loxP-mediated excision of *Bdnf* have led to the conclusion that *in vivo* the effects of endogenous BDNF in modulating the structure of neurons seem to be extremely specific, depending on the developmental stage, the brain area, as well as the cell-type. Another level of complexity lies in the multiple downstream signalling cascades, as well as the diametrically opposing effects of the pro- and mature forms which act through distinct receptors, tropomyosin-like kinase B (TrkB) and p75 (neurotrophin receptor, NTR) respectively.

The synergistic interactions between neuronal activity and synaptic plasticity induced by BDNF make it an ideal and essential regulator of cellular processes that underlie cognition and other complex behaviours. Indeed, numerous studies have firmly established that BDNF plays a critical role in hippocampal long-term potentiation (LTP), a long-term enhancement of synaptic efficacy implicated in learning and memory. Converging evidence now strongly implies that deficits in BDNF signalling contribute to the pathogenesis of several major diseases and disorders, such as Huntington’s disease, Alzheimer’s disease, and depression. Thus, manipulating BDNF pathways represents a viable therapeutic approach for a variety of neurological and psychiatric disorders.

Several recent reports suggest a link between neurotrophins, neuronal development and phospholipase D (PLD) activity. For example PLD1 regulates basic fibroblast growth factor (bFGF)-induced neurotrophin-3 expression and neurite outgrowth in immortalized hippocampal progenitor cells^9^,^10^,^11^,^12^. We have recently shown that cortical neurons cultured from mice lacking *Pld1* exhibit a significant delay in growth and development^13^. In line with this observation, *Pld1* knockout mice display impaired brain development and reduced cognitive function up to one month of age^14^. Furthermore, we found that neuronal growth factor (NGF)-induced neurite outgrowth requires phosphorylation of PLD1 by the serine-threonine kinase RSK2 in PC12 cells, and that the production of phosphatidic acid facilitates exocytosis of vesicle-associated membrane protein (VAMP)-7 vesicles at growth cones^13^. Interestingly the phosphorylation site for RSK2 is not found in PLD2, suggesting that this pathway is specific for PLD1 signalling^15^. A loss of function mutation in *RSK2* is responsible for the Coffin–Lowry syndrome (CLS), a rare syndromic form of mental retardation (MR) that shows X-linked inheritance^16^. These data suggest that the loss of RSK2 leading to CLS and neuronal deficits is related to defects in neuronal growth due to impaired RSK2-dependent PLD1 activity following NGF stimulation. Here, we investigated whether PLD1 is directly involved in BDNF signalling through a process involving RSK2 and found that PLD1 contributes to the regulation of multiple intracellular signalling cascades, including retrograde messages relying on vesicular PEA15 complex.

## Material and Methods

### Materials

Antibodies anti-HA (Babco), anti-RSK2, anti-APPL1, anti-Rab7, anti-TrkB (Santa Cruz Bio-technology), anti-PLD1, anti-ERK, anti-phospho-ERK, (New England BioLabs), anti-β-tubulin, anti-CREB (Millipore), anti-GAPDH, anti-phospho-CREB (Ser-133), anti-mTOR, anti-phospho-mTOR (Ser-2481), anti-phospho-S6K (Thr-389), anti-phospho-S6K (Thr-421/Ser-424), anti-PEA15 (Cell Signalling), anti-Rab5 (Transduction Laboratories) were used. Plasmids have been described previously^15^,^17^. ON-TARGETplus siRNA were obtained from Darmacon and BDNF was from Invitrogen.

### PLD assay

WT and *Rsk2*^*y/−*^ cortical neurons from E17 mice were plated at 40,000 cells per well and at 3 DIV were incubated for increasing time with 100 ng/mL of BDNF and used to measure PLD activity as described previously^18^. Briefly cells were washed twice with PBS and medium was then replaced by 100 μl of an ice-cold Tris 50 mM pH 8.0 solution and the cells broken by three freeze and thaw cycles. Samples were collected, mixed with an equal amount of the Amplex Red reaction buffer (Amplex Red Phospholipase D assay kit, Molecular Probes, USA) and the PLD activity estimated after 1 h incubation at 37 °C with a Mithras (Berthold) fluorimeter. A standard curve was performed with purified PLD from Streptomyces chromofuscus (Sigma). Data are normalized to the activity in WT neurons in the absence of treatment.

### Animals, Cell Culture and BDNF treatment

*Pld1*^−/−^ and *Rsk2*^y/−^ mice have been described previously^19^,^20^. They were housed and raised at Chronobiotron UMS 3415. All experiments were carried out in accordance with the European Communities Council Directive of 24^th^ November 1986 (86/609/EEC) and resulting French regulations. Accordingly all experimental protocol were approved by the CREMEAS local ethical committee. Every effort was made to minimize the number of animals used and their suffering. Cortical neurons were cultured from E17 mice in Neurobasal medium (Invitrogen) supplemented with B-27, 1% GlutaMAX and 1% Pen/Strep. Low-density cultures were plated on poly-L-lysine (Sigma)-coated glass coverslips at 25000 neurons/cm^2^. BDNF was applied to cells 72 h after transfection at 100 ng/mL for the indicated times.

### Transfection

Cultured cortical neurons were transfected at 2 DIV with Lipofectamine 2000 (Invitrogen) according to the manufacture’s instruction and analysed between 3 and 6 DIV. Transfection efficiency for HA- and GFP-tagged protein ranged between 15 and 25%.

### Western Blot and Immunoprecipitation

After treatment, cells were lysed and proteins were resolved by SDS 4–12% PAGE. Proteins were transferred to nitrocellulose membranes as previously described (de Barry *et al.*, 2006). Detection was performed by chemiluminescence using the Super Signal West Dura Extended Duration Substrate (Pierce). For immunoprecipitations, protein extracts were prepared in lysis buffer (50 mM Hepes, 3 mM EGTA, 3 mM CaCl_2_, 3 mM MgCl_2_, 80 mM KCl, 0.1% Triton X-100, 0.1% sodium deoxycholate, 1 mM sodium orthovanadate, 40 mM NaF, and protease inhibitor mixture (Sigma-Aldrich)). Five hundred micrograms of total protein were used. Quantification of Western blots was performed as described earlier^15^.

### Immunocytochemistry

Neurons were fixed and further processed for immunofluorescence as described previously^21^. Stained cells were visualized with a Leica SP5II confocal microscope. Quantification of co-localization was performed using Image J (JaCob plugin). Nuclear levels of phospho-ERK and phospho-CREB staining were estimated by the ratio: fluorescence intensity/the nucleus area, the later being estimated by DAPI staining. For imaging analysis at least 100 cells were analysed for each condition obtained from at least two independent cell cultures.

### Statistics

Number of samples, cell analysed and repeats are indicated in the figure legends. Statistical analysis was performed with R software using parametric and non parametric t-tests compared to the corresponding control condition (*p < 0.05; **p < 0.01; ***p < 0.001).

## Results

### RSK2 is required for neuronal PLD1 activation by BDNF-

**Figure 1:**
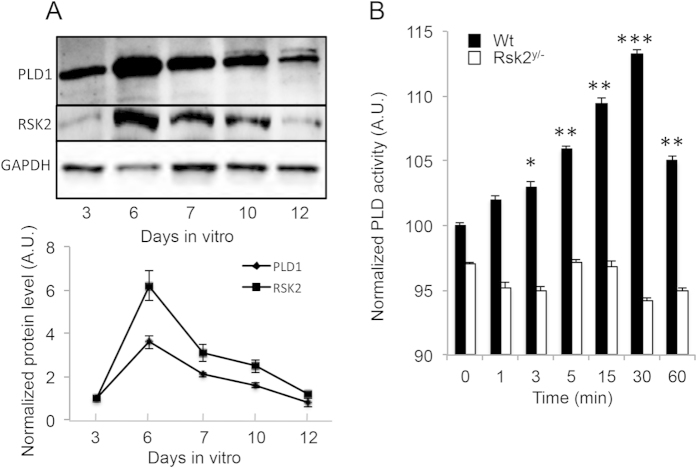
PLD1 and RSK2 expression and PLD activity in cultured cortical neurons. (**A**) E17 cortical neurons from control C57BL6 mice were cultured and lyzed between 3 and 12 DIV. 35 μg of proteins in each condition was resolved by SDS-PAGE and probed with anti-PLD1, anti-RSK2 and anti-GAPDH antibodies. The expression levels of PLD1 and RSK2 were quantified for two independent experiments and normalized to GAPDH levels. (**B**) WT and *Rsk2*^−/−^ cortical neurons at 3 DIV were incubated for 1 to 60 minutes with 100 ng/mL of BDNF and used to measure PLD activity. Data are normalized to the activity in WT neurons in the absence of treatment and were obtained from two independent measurements with sextuplicates.

Using cultures E17 mouse cortical neurons, we observed that RSK2 and PLD1 are highly expressed during the first week of culture, with expression levels for both proteins being maximal at 6 days *in vitro* (DIV) (Fig. 1A). Interestingly, formation and development of neuronal dendrites occurred between 4 and 15 DIV, suggesting that the activity of RSK2 and PLD1 could be involved in this process, in agreement with our recent finding that PLD1 KO neurons have less complex arborisation^13^. At earlier time (3DIV) when expression levels of PLD1 are submaximal, BDNF induced a time-dependent increase in PLD activity in cortical neuron cultures with a maximal effect measured after 30 min of stimulation (Fig. 1B). On the other hand, BDNF failed to trigger PLD activity in *Rsk2*^*y/−*^ neurons, suggesting that RSK2 may be an essential element in the signalling cascade that leads to BDNF-induced PLD activation.

### PLD1 regulates the ERK1/2-CREB and mTor-S6K signalling pathways in response to BDNF-

**Figure 2:**
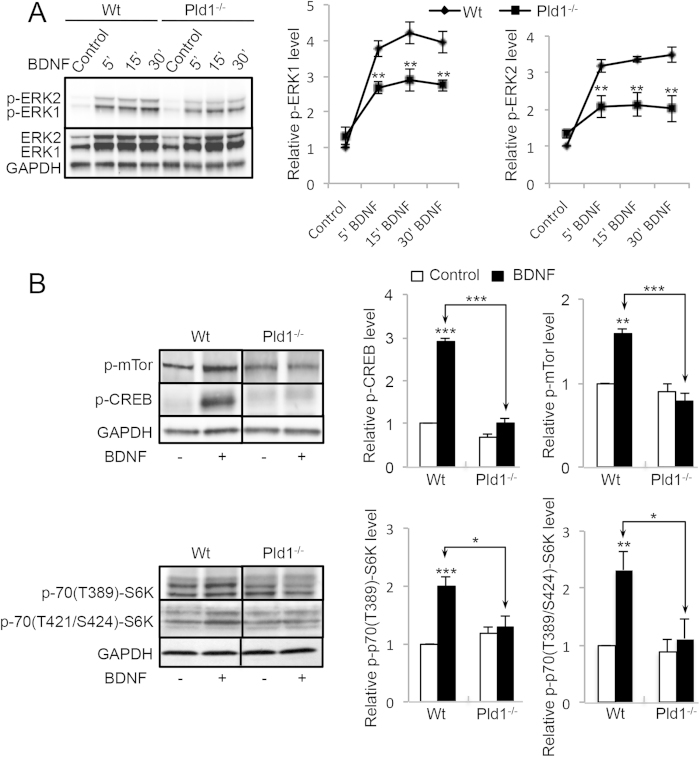
PLD1 is involved in different signalling pathway triggered by BDNF in cultured cortical neurons. (**A**) WT and *Pld1*^−/−^ cortical neurons in culture for 3 DIV were treated for the indicated time with 100 ng/mL of BDNF and lyzed. 35 μg of proteins was resolved by SDS-PAGE and probed with anti-ERK1/2, anti-phospho-ERK1/2 and anti-GAPDH antibodies. The phosphorylation level of ERK1/2 was corrected to ERK1/2 expression levels and subsequently normalized to GAPDH expression levels. The relative ERK1 and ERK2 is normalized to that of WT untreated cells obtained from three independent experiments. (**B**) WT and *Pld1*^−/−^ cortical in culture for 3 DIV were treated for 15 minutes with 100 ng/mL of BDNF and lyzed. 35 μg of proteins was resolved by SDS-PAGE and probed with anti-phospho-mTor (Ser-2481), anti-phospho-S6K (Thr-389), anti-phospho-CREB (Ser-133) and anti-GAPDH antibodies. The levels of phospho-proteins was normalized to GAPDH expression levels and normalized to that of WT untreated cells. For pS6K quantification, lower bands levels corresponding to P70S6K were analysed. Data were averaged from three independent experiments.

BDNF stimulation of cortical neurons induced a rapid phosphorylation of ERK1/2 that reached a maximum near 15 minutes of treatment (Fig. 2A). In the absence of PLD1, the phosphorylation levels of ERK1/2 (p-p42 and p-p44) after BDNF treatment were significantly reduced (Fig. 2A), suggesting that PLD1 plays a positive role in the optimal activation of the ERK signalling pathway in response to BDNF. BDNF also induced a strong increase in phospho-CREB levels (p-CREB), an effect that was almost completely abolished in *Pld1*^−/−^ neurons (Fig. 2B), in line with the idea that PLD1 activity contributes to the ERK-CREB signalling pathway. At the same time, we found that PLD1 is required for *optimal* BDNF-induced phosphorylation of mTor (p-mTor) and S6Kinase (both for p-70(389Thr)-S6K and p-70(421Thr/Ser424)-S6K) (Fig. 2B), suggesting that PLD1 also affects this signalling pathways.

### PLD1 overexpression modulates the nuclear level of p-ERK1/2 and p-CREB-

**Figure 3:**
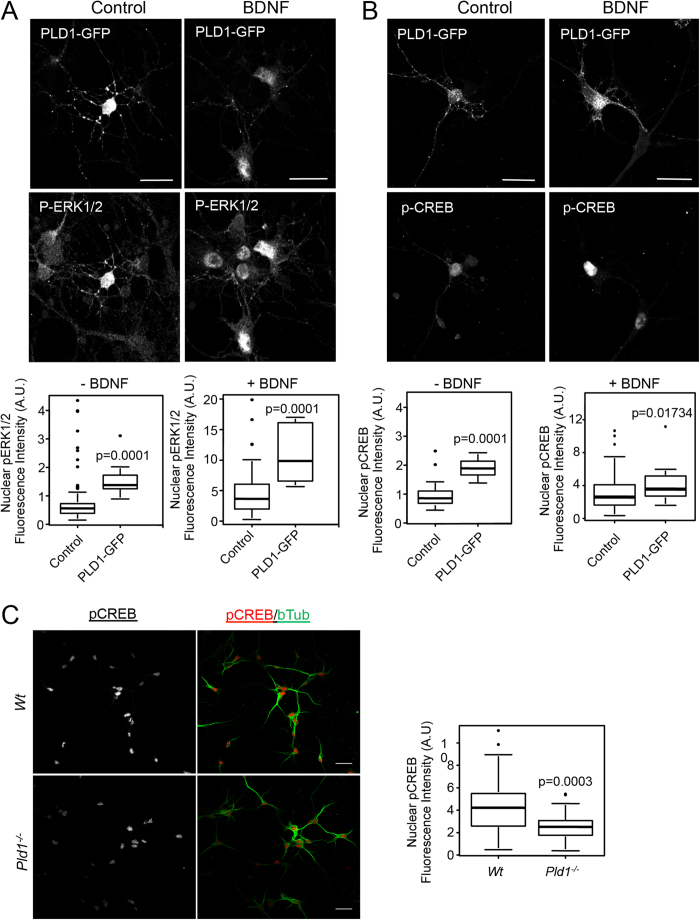
PLD1 regulates the nuclear level of p-ERK1/2 and p-CREB. 2 DIV WT cortical neurons were transfected with PLD1-GFP and after 24 hours treated for 15 minutes with 100 ng/mL of BDNF. After fixation cells were stained with anti-phospho-ERK1/2 (**A**) or anti-phospho-CREB (**B**) antibodies and the nucleus stained with DAPI (not shown). (**C**) WT and *Pld1*^−/−^ cortical neurons in culture for 3 DIV were treated for 15 minutes with 100 ng/mL of BDNF. After fixation cells were stained with anti-phospho-CREB and anti-β-tubulin antibodies, and the nucleus was stained with DAPI (not shown). The level of nuclear phospho-ERK1/2 (**A**) or phospho-CREB (**B**,**C**) fluorescence intensity was normalized to the nuclear area revealed by DAPI staining from at least 30 cells per condition. Bar = 25 μm.

Since BDNF has been reported to affect the nuclear levels of p-ERK1/2 and the transcription factor p-CREB^22^,^23^, we next tested whether PLD1 is involved in this part of the ERK-CREB pathway. Although in Wt and *Pld1*^−/−^ neurons, nuclearp-ERK1/2 staining after BDNF treatment was only modestly and non-significantly reduced (data not shown), the overexpression of PLD1-GFP nearly tripled the nuclear p-ERK1/2 signal in both control and BDNF-treated neurons (Fig. 3A). Of note, we also observed that BDNF treatment strongly increased the intensity of nuclear p-ERK1/2 signal, an effect that was potentiated in cells overexpressing PLD1-GFP (Fig. 3A). Similarly, the overexpression of PLD1-GFP approximately doubled the nuclear p-CREB signal in control and BDNF treated cells (Fig. 3B). Interestingly the level of p-CREB signal in BDNF treated neurons was reduced by nearly 50% in the absence of PLD1 (Fig. 3C). All together these results suggest that PLD1 may contribute to the signalling pathway that leads to increased levels of p-ERK1/2 and p-CREB in the nucleus of cortical neurons after BDNF stimulation.

### PLD1, p-ERK1/2 and RSK2 on partially associated with vesicular structures-

**Figure 4:**
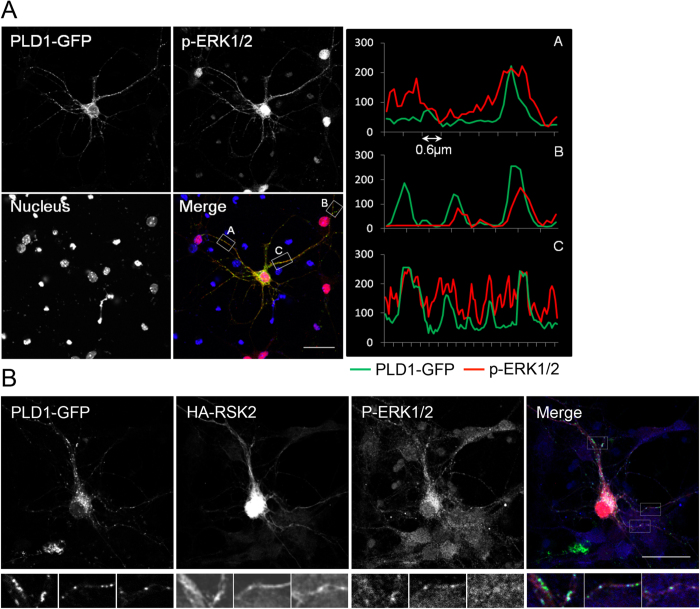
PLD1 colocalizes with p-ERK1/2 and RSK2 on vesicular structures. 2 DIV WT cortical neurons were transfected with PLD1-GFP (**A**) or PLD1-GFP and HA-RSK2 and 24 hours later, treated for 15 minutes with 100 ng/mL BDNF. After fixation, cells were stained with anti-phospho-ERK1/2 and/or with anti-HA antibodies. Selected regions of interest revealed partial colocalization of PLD1-GFP with phospho-ERK1/2 (**A**) and of PLD1-GFP with HA-RSK2, and with phospho-ERK1/2 (**B**). Similar observations were obtained from two independent cultures. Bars = 25 μm.

**Figure 5:**
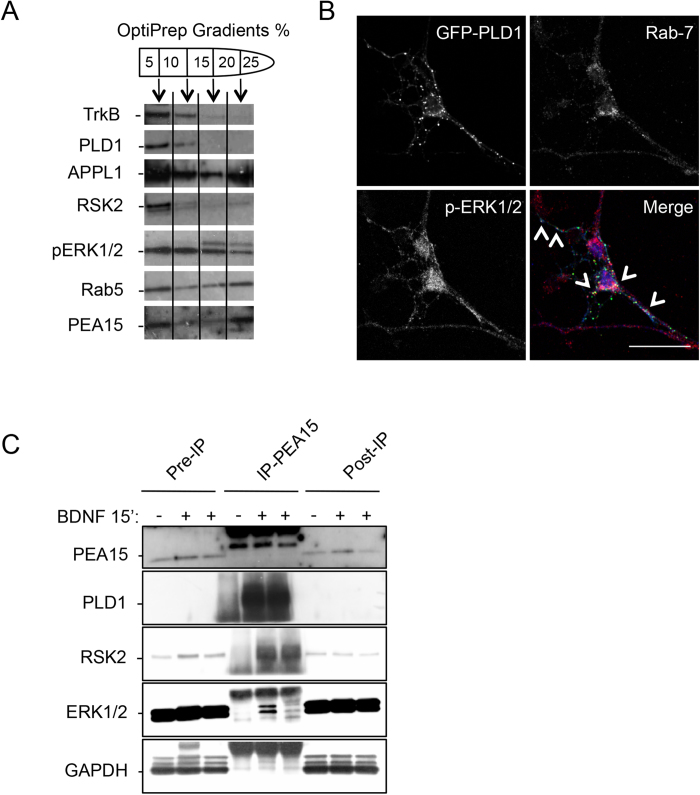
PLD1, p-ERK1/2 and PEA15 are found on late endosomal vesicles. (**A**) 3 DIV neurons treated for 15 minutes with 100 ng/mL of BDNF were lyzed and subjected to subcellular fractionation by velocity centrifugation on OptiPrep gradient (5-10-15-20-25%). Fractions were collected and after Western blotting probed with anti-TrkB, anti-PLD1, anti-APPL1, anti-RSK2, anti-pERK1/2, anti-Rab5, and anti-PEA15 antibodies. Similar observations were obtained on two independent experiments. (**B**) 2 DIV WT cortical neurons were transfected with PLD1-GFP and 24 hours later treated for 15 minutes with 100 ng/mL BDNF. After fixation, cells were stained with anti-phospho-ERK1/2 and anti-Rab7 antibodies. Arrows indicate vesicular structures positives for PLD1, Rab7, and phospho-ERK1/2. Bar = 25 μm. (**C**) 3 DIV neuronal cultures untreated (−) or treated ( + ) for 15 minutes with 100 ng/mL BDNF were lyzed, and PEA15 was immunoprecipitated. Pre-immunoprecipitation, immunoprecipitated and post-immuniprecipitated samples were probed with anti-PEA15, anti-PLD1, anti-RSK2, anti-ERK1/2, and anti-GAPDH antibodies. GAPDH was used a negative control of immuonprecipitation. Note that the amount of PEA15 immunoprecipitated after BDNF treatment varied and that the amount of ERK1/2 coimmunoprecipitated varied accordingly. Similar observations were replicated twice.

Having shown that RSK2 regulates PLD activation in response to BDNF treatment and that PLD1 contributes to the activation of the ERK1/2-CREB signalling pathway the localization of PLD1, RSK2, and phospho-ERK1/2 was examined. However due to the lack of specific PLD1 or RSK2 antibodies capable to detect endogenous proteins for immunofluorescence experiments we overexpressed tagged versions of the proteins. After 15 minutes of BDNF treatment, 67.5% ± 2.3% of the GFP-PLD1 signal colocalized with phospho-ERK1/2 mainly in the perinuclear area, but also in dendrites and axons (Fig. 4A). Analysis of the fluorescence in the cellular extensions indicated that both staining were associated on punctuate structures (Fig. 4A). BDNF treatment of neurons overexpressing HA-RSK2 and GFP-PLD1 revealed that both proteins partially colocalized with p-ERK1/2 on vesicular structures (Fig. 4B). In subcellular fractions of BDNF-treated obtained using an OptiPrep gradient, endogenous PLD1 and RSK2 were mostly enriched at the 5–10% interphase, along with the BDNF receptor TrkB and the early endosomal markers, APP1 and Rab5 (Fig. 5A). Significant amounts of pERK1/2 were also present in this fraction (Fig. 5A), in agreement with previous findings^24^.

Interestingly the phosphoprotein PEA15 was highly enriched in the upper gradient endosomal fraction purified from BDNF treated neurons (Fig. 5A). PEA15, predominantly expressed in the central nervous system has been recently shown to have binding sites for PLD1, RSK2 and ERK1/2^25^,^26^,^27^,^28^, suggesting that it could be an ideal hub to integrate these signalling molecules. In line with the notion that these signalling molecules may transport the information towards the nucleus through the endocytic pathway, we also found that overexpressed GFP-PLD1 partially colocalized with pERK1/2 on late endosomal Rab7-positive vesicles after BDNF treatment (Fig. 5B).

### PEA15 is required for the BDNF-induced increase in the nuclear level of p-ERK1/2 and p-CREB-

**Figure 6:**
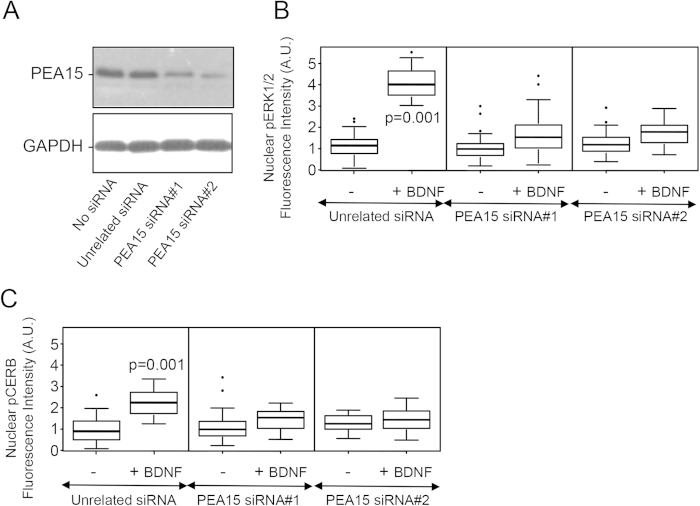
Effect of PEA15 silencing on BDNF-induced increases in the nuclear level of p-ERK1/2 and p-CREB. (**A**) 2 DIV WT cortical neurons were transfected with two different siRNA for PEA15, an unrelated siRNA, or mock-transfected. After 72 h, cells were lysed and 35 μg of proteins was resolved by SDS-PAGE and probed with anti-PEA15 and anti-GAPDH antibodies. Similar results were obtained in two independent experiments. (**B**,**C**) Cortical neurons expressing either the unrelated siRNA, or PEA15-siRNA#1, or PEA15-siRNA#2, were untreated (−) or treated ( + ) for 15 minutes with 100 ng/mL of BDNF. After fixation, cells were stained with anti-phospho-ERK1/2 or anti-phospho-CREB antibodies and the nucleus stained with DAPI (not shown). The level of nuclear phospho-ERK1/2 (**B**) or phospho-CREB (**C**) fluorescence intensity was normalized to the nuclear area revealed by DAPI staining.

Using an immunoprecipitation strategy, endogenous PLD1, RSK2 and ERK1/2 were coprecipitated with endogenous PEA15, in agreement with the idea that these proteins form a large complex in cortical neurons in response to BDNF treatment (Fig. 5C). To probe the notion that PEA15 may contribute to the signalling pathway that triggers elevated phospho-ERK and phospho-CREB levels in the nucleus, the endogenous level of PEA15 was reduced with small interference RNA. Hence, PEA15 levels were reduced by 73 ± 8% with siRNA#1 and by 86 ± 5% with siRNA#2 (Fig. 6A). Although reduced PEA15 expression had no effect on the nuclear levels of p-ERK1/2 and p-CREB in untreated neurons, it almost completely abolished the enhanced nuclear levels of these proteins in the response to BDNF (Fig. 6B,C). Altogether these results suggest that PEA15 is necessary to convoy the PLD1-RSK2-ERK signal towards the nucleus of neurons.

## Discussion

Neurotrophins, including BDNF are key regulators of synaptic function and neuronal development^29^,^30^. Neurotrophins signal by binding and activating receptors with intrinsic tyrosine kinase activity. It is now well accepted that BDNF promotes neuronal development and synaptic plasticity through multiple signalling and protein synthesis-dependent mechanisms that differ between brain regions, cell types, and probably as a function of the type of induced-LTP^31^. BDNF is crucially involved in nearly all developmental stages of neuronal circuitry including, i) survival of stem cells and progenitors, ii) neurogenesis and neuronal differentiation, iii) neuronal polarization and guidance, iv) branching and survival of differentiated neurons, and v) formation and maturation of spines and synapses^32^,^33^,^34^,^35^,^36^,^37^,^38^,^39^. Currently, the dynamics of BDNF-TrkB signalling and its impact on downstream signalling events are not well understood. Using cortical neuron cultures, we show here that PLD1 expression is required for phosphorylation of ERK1/2, CREB, mTor, and S6K in an acute response to BDNF. This result is in agreement with the well-known involvement of the ERK1/2-RSK2-CREB and mTor-p70/S6K pathways as major regulators of neuronal morphology and plasticity^40^,^41^, but also with the signalling pathways downstream of PLD, although in different cell type and with different agonist^42^,^43^. In line with a critical role of PLD1 during neuronal development, PLD1 expression was found to dramatically increase in cortical neurons after 3 days in culture, as did RSK2 expression level. BDNF treatment induced a rapid (within minutes) and transient activation of PLD activity in cultured neurons that was not seen in *Rsk2*^y/−^ neurons, arguing that RSK2 acts upstream of PLD1. Having previously shown that RSK2 can phosphorylate PLD1 on Thr147 during neurosecretion^15^, one can speculate that RSK2 phosphorylation of PLD1 is also required for the activation of PLD in cortical neurons treated with BDNF. Of note, in lysates of cortical neurons cultured for 10 and 12 DIV a second higher molecular weight band positive for PLD1 that may correspond to a phosphorylated form of PLD1 was detected. This could, however, not be tested experimentally because the anti-PLD1-phospho-Thr147 antibody doe not recognize the mouse phospho-PLD1 (data not shown). In line with this possibility, PLD1 was recently found in a screen of phosphorylated proteins in mouse synaptosomes^44^.

The BDNF-activated intracellular signal transduction pathways probably mediate neuronal adaptation, in part by modifying existing proteins through phosphorylation for instance, but also by activating transcription factors that regulate gene expression^45^. However the signal transduction pathways that connect signals generated at synapses with transcriptional responses in the nucleus are not well understood. In the present report, BDNF treatment increased the nuclear accumulation of phospho-ERK1/2 and phospho-CREB in a PLD1-dependent manner. Furthermore, PLD1, RSK2, and ERK1/2 were found colocalized on vesicular structures present both in neuronal axons and dendrites. These structures were positive for Rab5, Rab7 and APP1, indicating that they are part of the recycling endosomal system. A model for the regulation of ERK1/2 phosphorylation by cell surface receptors involves Ras–Raf complexes bound to the surface of endosomes, where scaffolding complexes involving Ras, cRaf-1, MEK and ERK1/2 are formed^46^. Complete activation and coupling of this cascade requires endocytosis, a process that is also modulated by phosphatidic acid (PA) produced by PLD^47^. Therefore, the current data suggest that RSK2 and PLD1 may also be part of this complex on recycling endosomes.

In line with the finding that the scaffolding protein PEA15 contains PLD1, RSK2, and ERK1/2 binding sites^48^, the present immunoprecipitation results validate the notion that BDNF triggers the formation of a multiprotein complex containing these four partners in cortical neurons. Furthermore, PEA15 silencing significantly affected the levels of nuclear phospho-ERK1/2 and phospho-CREB, providing evidence that this quadripartite complex is probably participates in the retrograde transport of the molecular modules involved in BDNF signalling.

Alterations in BDNF levels are associated with neurodegenerative disorders (including Alzheimer’s disease, Huntington’s disease and epilepsy), neuropsychiatric disorders (including depression, anxiety disorders, bipolar disorders, schizophrenia and addiction) and obesity^49^,^50^. The hallmark of BDNF deficiency is synaptic degeneration, whereas increasing BDNF levels promote synaptic repair in preclinical models^49^,^51^,^52^. Moreover, BDNF could potentially be used to treat diseases in which alterations in its levels are not directly involved in the pathogenesis (for instance, in Parkinson’s disease, amyotrophic lateral sclerosis, stroke and spinal cord injury). BDNF is a highly charged protein that does not readily cross the blood–brain barrier making CNS delivery a challenge. So targeting molecules involved in BDNF signalling may represent alternative strategies. The current data indicating a role for PLD1 in BDNF signalling opens up the possibility to compensate decreased or increased PLD1 signalling/activity in this pathway. The novel small molecule inhibitors for PLD may represent interesting candidates in the case that increased PLD1 activity could affect BDNF signalling in a pathophysiological context. This is especially appealing since several reports have implicated PLD in neurodegenerative disorders (reviewed in^43^).

## Additional Information

**How to cite this article**: Ammar, M. R. *et al.* PLD1 participates in BDNF-induced signalling in cortical neurons. *Sci. Rep.* **5**, 14778; doi: 10.1038/srep14778 (2015).

## References

[b1] Gottmann K., Mittmann T. & Lessmann V. BDNF signaling in the formation, maturation and plasticity of glutamatergic and GABAergic synapses. Exp Brain Res 199, 203–234, 10.1007/s00221-009-1994-z (2009). 19777221

[b2] Park H. & Poo M. M. Neurotrophin regulation of neural circuit development and function. Nat Rev Neurosci 14, 7–23, 10.1038/nrn3379 (2013). 23254191

[b3] Zagrebelsky M. & Korte M. Form follows function: BDNF and its involvement in sculpting the function and structure of synapses. Neuropharmacology 76 Pt C, 628–638, 10.1016/j.neuropharm.2013.05.029 (2014). 23752094

[b4] McAllister A. K. Spatially restricted actions of BDNF. Neuron 36, 549–550 (2002). 12441043 10.1016/s0896-6273(02)01063-2

[b5] Horch H. W. & Katz L. C. BDNF release from single cells elicits local dendritic growth in nearby neurons. Nat Neurosci 5, 1177–1184, 10.1038/nn927 (2002). 12368805

[b6] Ji Y. *et al.* Acute and gradual increases in BDNF concentration elicit distinct signaling and functions in neurons. Nat Neurosci 13, 302–309, 10.1038/nn.2505 (2010). 20173744 PMC4780419

[b7] Ji Y., Pang P. T., Feng L. & Lu B. Cyclic AMP controls BDNF-induced TrkB phosphorylation and dendritic spine formation in mature hippocampal neurons. Nat Neurosci 8, 164–172, 10.1038/nn1381 (2005). 15665879

[b8] Hong E. J., McCord A. E. & Greenberg M. E. A biological function for the neuronal activity-dependent component of Bdnf transcription in the development of cortical inhibition. Neuron 60, 610–624, 10.1016/j.neuron.2008.09.024 (2008). 19038219 PMC2873221

[b9] Choi H. J., Chang B. J. & Han J. S. Phospholipase D1 is an important regulator of bFGF-induced neurotrophin-3 expression and neurite outgrowth in H19-7 cells. Mol Neurobiol 45, 507–519, 10.1007/s12035-012-8268-7 (2012). 22544632

[b10] Yoon M. S. *et al.* Role of phospholipase D1 in neurite outgrowth of neural stem cells. Biochem Biophys Res Commun 329, 804–811, 10.1016/j.bbrc.2005.02.087 (2005). 15752728

[b11] Klein J. Functions and pathophysiological roles of phospholipase D in the brain. J Neurochem 94, 1473–1487, 10.1111/j.1471-4159.2005.03315.x (2005). 16042758

[b12] Zhang Y., Kanaho Y., Frohman M. A. & Tsirka S. E. Phospholipase D1-promoted release of tissue plasminogen activator facilitates neurite outgrowth. J Neurosci 25, 1797–1805, 10.1523/JNEUROSCI.4850-04.2005 (2005). 15716416 PMC6725938

[b13] Ammar M. R. *et al.* The Coffin-Lowry syndrome-associated protein RSK2 regulates neurite outgrowth through phosphorylation of phospholipase D1 (PLD1) and synthesis of phosphatidic acid. J Neurosci 33, 19470–19479, 10.1523/JNEUROSCI.2283-13.2013 (2013). 24336713 PMC6618760

[b14] Burkhardt U. *et al.* Impaired brain development and reduced cognitive function in phospholipase D-deficient mice. Neurosci Lett 572, 48–52, 10.1016/j.neulet.2014.04.052 (2014). 24813107

[b15] Zeniou-Meyer M. *et al.* The Coffin-Lowry syndrome-associated protein RSK2 is implicated in calcium-regulated exocytosis through the regulation of PLD1. Proc Natl Acad Sci USA 105, 8434–8439, 10.1073/pnas.0710676105 (2008). 18550821 PMC2448854

[b16] Hanauer A. & Young I. D. Coffin-Lowry syndrome: clinical and molecular features. J Med Genet 39, 705–713 (2002). 12362025 10.1136/jmg.39.10.705 PMC1734994

[b17] Zeniou-Meyer M. *et al.* Phospholipase D1 production of phosphatidic acid at the plasma membrane promotes exocytosis of large dense-core granules at a late stage. J Biol Chem 282, 21746–21757, 10.1074/jbc.M702968200 (2007). 17540765

[b18] Lopez C. I. *et al.* Diacylglycerol stimulates acrosomal exocytosis by feeding into a PKC- and PLD1-dependent positive loop that continuously supplies phosphatidylinositol 4,5-bisphosphate. Biochimica et biophysica acta 1821, 1186–1199, 10.1016/j.bbalip.2012.05.001 (2012). 22609963

[b19] Elvers M. *et al.* A novel role for phospholipase D as an endogenous negative regulator of platelet sensitivity. Cell Signal 24, 1743–1752, 10.1016/j.cellsig.2012.04.018 (2012). 22579635

[b20] Yang X. *et al.* ATF4 is a substrate of RSK2 and an essential regulator of osteoblast biology; implication for Coffin-Lowry Syndrome. Cell 117, 387–398 (2004). 15109498 10.1016/s0092-8674(04)00344-7

[b21] Vitale N., Moss J. & Vaughan M. Molecular characterization of the GTPase-activating domain of ADP-ribosylation factor domain protein 1 (ARD1). The Journal of biological chemistry 273, 2553–2560 (1998). 9446556 10.1074/jbc.273.5.2553

[b22] Zhou X., Xiao H. & Wang H. Developmental changes of TrkB signaling in response to exogenous brain-derived neurotrophic factor in primary cortical neurons. J Neurochem 119, 1205–1216, 10.1111/j.1471-4159.2011.07528.x (2011). 21988201 PMC3230766

[b23] Kim J. H. *et al.* Brain-derived neurotrophic factor uses CREB and Egr3 to regulate NMDA receptor levels in cortical neurons. J Neurochem 120, 210–219, 10.1111/j.1471-4159.2011.07555.x (2012). 22035109 PMC5116917

[b24] Liu W. *et al.* Establishment of extracellular signal-regulated kinase 1/2 bistability and sustained activation through Sprouty 2 and its relevance for epithelial function. Molecular and cellular biology 30, 1783–1799, 10.1128/MCB.01003-09 (2010). 20123980 PMC2838067

[b25] Zhang Y. *et al.* Regulation of expression of phospholipase D1 and D2 by PEA-15, a novel protein that interacts with them. J Biol Chem 275, 35224–35232, 10.1074/jbc.M003329200 (2000). 10926929

[b26] Hill J. M., Vaidyanathan H., Ramos J. W., Ginsberg M. H. & Werner M. H. Recognition of ERK MAP kinase by PEA-15 reveals a common docking site within the death domain and death effector domain. EMBO J 21, 6494–6504 (2002). 12456656 10.1093/emboj/cdf641 PMC136945

[b27] Vaidyanathan H. & Ramos J. W. RSK2 activity is regulated by its interaction with PEA-15. J Biol Chem 278, 32367–32372, 10.1074/jbc.M303988200 (2003). 12796492

[b28] Vaidyanathan H. *et al.* ERK MAP kinase is targeted to RSK2 by the phosphoprotein PEA-15. Proc Natl Acad Sci USA 104, 19837–19842, 10.1073/pnas.0704514104 (2007). 18077417 PMC2148384

[b29] Schuman E. M. Neurotrophin regulation of synaptic transmission. Curr Opin Neurobiol 9, 105–109 (1999). 10072368 10.1016/s0959-4388(99)80013-0

[b30] Bramham C. R. & Panja D. BDNF regulation of synaptic structure, function, and plasticity. Neuropharmacology 76 Pt C, 601–602, 10.1016/j.neuropharm.2013.08.012 (2014). 23973290

[b31] Panja D. & Bramham C. R. BDNF mechanisms in late LTP formation: A synthesis and breakdown. Neuropharmacology 76 Pt C, 664–676, 10.1016/j.neuropharm.2013.06.024 (2014). 23831365

[b32] Huang E. J. & Reichardt L. F. Trk receptors: roles in neuronal signal transduction. Annu Rev Biochem 72, 609–642, 10.1146/annurev.biochem.72.121801.161629 (2003). 12676795

[b33] Minichiello L. *et al.* Mechanism of TrkB-mediated hippocampal long-term potentiation. Neuron 36, 121–137 (2002). 12367511 10.1016/s0896-6273(02)00942-x

[b34] Lu B. BDNF and activity-dependent synaptic modulation. Learn Mem 10, 86–98, 10.1101/lm.54603 (2003). 12663747 PMC5479144

[b35] Xu B. *et al.* The role of brain-derived neurotrophic factor receptors in the mature hippocampus: modulation of long-term potentiation through a presynaptic mechanism involving TrkB. J Neurosci 20, 6888–6897 (2000). 10995833 10.1523/JNEUROSCI.20-18-06888.2000 PMC2711895

[b36] Gomes R. A., Hampton C., El-Sabeawy F., Sabo S. L. & McAllister A. K. The dynamic distribution of TrkB receptors before, during, and after synapse formation between cortical neurons. J Neurosci 26, 11487–11500, 10.1523/JNEUROSCI.2364-06.2006 (2006). 17079678 PMC6674530

[b37] McAllister A. K. Bdnf. Curr Biol 12, R310 (2002). 12007428 10.1016/s0960-9822(02)00825-4

[b38] McAllister A. K., Katz L. C. & Lo D. C. Opposing roles for endogenous BDNF and NT-3 in regulating cortical dendritic growth. Neuron 18, 767–778 (1997). 9182801 10.1016/s0896-6273(00)80316-5

[b39] McAllister A. K., Lo D. C. & Katz L. C. Neurotrophins regulate dendritic growth in developing visual cortex. Neuron 15, 791–803 (1995). 7576629 10.1016/0896-6273(95)90171-x

[b40] Jaworski J., Spangler S., Seeburg D. P., Hoogenraad C. C. & Sheng M. Control of dendritic arborization by the phosphoinositide-3′-kinase-Akt-mammalian target of rapamycin pathway. J Neurosci 25, 11300–11312, 10.1523/JNEUROSCI.2270-05.2005 (2005). 16339025 PMC6725892

[b41] Kumar V., Zhang M. X., Swank M. W., Kunz J. & Wu G. Y. Regulation of dendritic morphogenesis by Ras-PI3K-Akt-mTOR and Ras-MAPK signaling pathways. J Neurosci 25, 11288–11299, 10.1523/JNEUROSCI.2284-05.2005 (2005). 16339024 PMC6725910

[b42] Foster D. A., Salloum D., Menon D. & Frias M. A. Phospholipase D and the maintenance of phosphatidic acid levels for regulation of mammalian target of rapamycin (mTOR). J Biol Chem 289, 22583–22588, 10.1074/jbc.R114.566091 (2014). 24990952 PMC4132766

[b43] Frohman M. A. The phospholipase D superfamily as therapeutic targets. Trends Pharmacol Sci 36, 137–144, 10.1016/j.tips.2015.01.001 (2015). 25661257 PMC4355084

[b44] Trinidad J. C. *et al.* Global identification and characterization of both O-GlcNAcylation and phosphorylation at the murine synapse. Molecular & cellular proteomics : MCP 11, 215–229, 10.1074/mcp.O112.018366 (2012). 22645316 PMC3412957

[b45] Stanton P. K. & Sarvey J. M. Blockade of long-term potentiation in rat hippocampal CA1 region by inhibitors of protein synthesis. J Neurosci 4, 3080–3088 (1984). 6502226 10.1523/JNEUROSCI.04-12-03080.1984 PMC6564864

[b46] Rizzo M. A., Shome K., Watkins S. C. & Romero G. The recruitment of Raf-1 to membranes is mediated by direct interaction with phosphatidic acid and is independent of association with Ras. The Journal of biological chemistry 275, 23911–23918, 10.1074/jbc.M001553200 (2000). 10801816

[b47] Andresen B. T., Rizzo M. A., Shome K. & Romero G. The role of phosphatidic acid in the regulation of the Ras/MEK/Erk signaling cascade. FEBS Lett 531, 65–68 (2002). 12401205 10.1016/s0014-5793(02)03483-x

[b48] Sulzmaier F. J. *et al.* PEA-15 potentiates H-Ras-mediated epithelial cell transformation through phospholipase D. Oncogene 31, 3547–3560, 10.1038/onc.2011.514 (2012). 22105357 PMC3295902

[b49] Lu B., Nagappan G. & Lu Y. BDNF and synaptic plasticity, cognitive function, and dysfunction. Handb Exp Pharmacol 220, 223–250, 10.1007/978-3-642-45106-5_9 (2014). 24668475

[b50] Rios M. BDNF and the central control of feeding: accidental bystander or essential player? Trends Neurosci 36, 83–90, 10.1016/j.tins.2012.12.009 (2013). 23333344 PMC3568936

[b51] Je H. S. *et al.* Role of pro-brain-derived neurotrophic factor (proBDNF) to mature BDNF conversion in activity-dependent competition at developing neuromuscular synapses. Proc Natl Acad Sci USA 109, 15924–15929, 10.1073/pnas.1207767109 (2012). 23019376 PMC3465384

[b52] Lu B., Nagappan G., Guan X., Nathan P. J. & Wren P. BDNF-based synaptic repair as a disease-modifying strategy for neurodegenerative diseases. Nat Rev Neurosci 14, 401–416, 10.1038/nrn3505 (2013). 23674053

